# Things are not always what they seem: From Cornelia de Lange to KBG phenotype in a girl with genetic variants in *NIPBL* and *ANKRD11*

**DOI:** 10.1002/mgg3.1826

**Published:** 2021-10-07

**Authors:** Ana Latorre‐Pellicer, Ángela Ascaso, Cristina Lucia‐Campos, Marta Gil‐Salvador, María Arnedo, Rebeca Antoñanzas, Ariadna Ayerza‐Casas, Iñigo Marcos‐Alcalde, Paulino Gómez‐Puertas, Feliciano J. Ramos, Juan Pié, Beatriz Puisac

**Affiliations:** ^1^ Unit of Clinical Genetics and Functional Genomics Department of Pharmacology‐Physiology School of Medicine Universidad de Zaragoza Zaragoza Spain; ^2^ Unit of Paediatric Cardiology Service of Paediatrics Hospital Universitario Miguel Servet Zaragoza Spain; ^3^ Molecular Modeling Group Centro de Biología Molecular Severo Ochoa CBMSO (CSIC‐UAM) Madrid Spain; ^4^ Biosciences Research Institute School of Experimental Sciences Universidad Francisco de Vitoria Madrid Spain; ^5^ Unit of Clinical Genetics Service of Paediatrics Hospital Clínico Universitario Lozano Blesa Department of Paediatrics School of Medicine Universidad de Zaragoza Zaragoza Spain

## Abstract

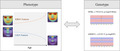

## CONFLICT OF INTEREST

The authors declare no competing interests.

## AUTHOR CONTRIBUTIONS

A.L.‐P., C.L.‐C., M.G.‐S., M.A., R.A., J.P., and B.P.: molecular analysis; A.A., A.A.‐C., and F.J.R.: patients’ recruitment, clinical evaluation, and clinical score calculation; I.M.‐A. and P.G.‐P.: bioinformatics studies and variants interpretation; A.L.‐P., J.P., and B.P.: manuscript writing, collection, and assembly of data; F.J.R., J.P., and B.P.: manuscript editing and approval of the manuscript. All authors have read and agreed to the submitted version of the manuscript.

## ETHICS APPROVAL

The employed procedure was reviewed and approved by the Ethics Committee of Clinical Research from the Government of Aragón (CEICA; PI15/00707). All human subjects participating in the research (or their legal guardians) signed the informed consent. An additional informed consent was collected and signed for the publication of subjects’ photographs.

Dear Editor,

The diagnosis success rates for developmental disorders have greatly improved in the last years mainly due to the widespread use of DNA next‐generation sequencing. Nevertheless, several studies have stressed the importance of a critical reconsideration of genetic results and a further implementation of protocols for variant‐level reevaluation and case‐level reanalysis (Deignan et al., 2019). This is especially relevant in the context of syndromes, such as the chromatinopathies Cornelia de Lange syndrome (CdLS, OMIM#122470) and KBG syndrome (KBGS, OMIM #148050), with overlapping phenotypes that may evolve over time (Parenti et al., 2021). Here, we present a challenging familiar case reanalyzed in which phenotypic features of both KBGS and CdLS are observed, and where genetic variants in *ANKRD11* and *NIPBL* were identified.

**FIGURE 1:**
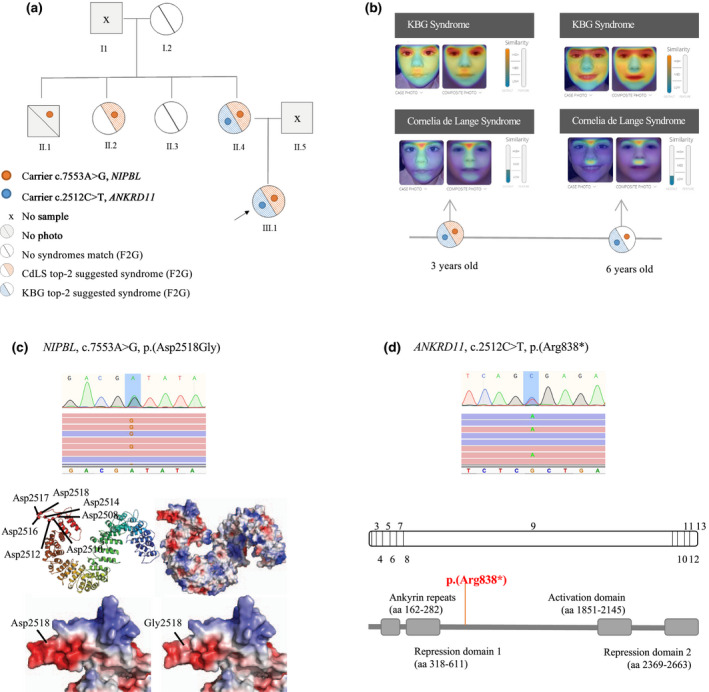
(a) Pedigree chart of the family showing Mendelian segregation of the heterozygous variants, c.7553A>G in *NIPBL* and c.2512C>T in *ANKRD11*, as well as the results of the Face2Gene (F2G) of the facial photographs of the individuals. (b) Face2Gene evaluation of facial photographs of the patient at 3 and 6 years of age, respectively. (c) Sanger chromatogram and Integrative Genomics Viewer (IGV) view of sequencing results of *NIPBL*: c.7553A>G in III.1, and structural modeling of NIPBL Asp2518Gly mutant. Top: predicted HEAT‐repeat arrangement of NIPBL residues 1538 to 2544. Position of negatively charged amino acids Asp2508, Asp2510, Asp2512, Asp2514, Asp2516, Asp2517. and Asp2518 is indicated. Bottom: mutation of Asp2518 to Gly promotes a decrease in the negative charge on the surface of the local patch. (d) Sanger chromatogram and Integrative Genomics Viewer (IGV) view of sequencing results of *ANKRD11*: c.2512C>T in III.1. Localization of the *ANKRD11* variant at protein and DNA levels

Our case is a female, first child of a non‐consanguineous couple, born at 37 weeks of gestation after an uneventful pregnancy. Birth length (47.2 cm, −0.55 SD), body weight (2.680 kg, −0.52 SD), and head circumference (34 cm, 0.26 SD) were all normal. She was referred to our hospital at the age of 3 years because of facial dysmorphism, gastroesophageal reflux, and motor developmental delay. After a comprehensive physical evaluation by our clinical geneticist, she was clinically diagnosed as CdLS with a clinical score of 10, mainly due to the synophrys (HP:0000664), thick eyebrows (HP:0000574), concave nasal ridge (HP:0011120), downturned corners of mouth (HP:0002714), global developmental delay (HP:0001263), small hands (HP:0200055) and feet (HP:0001773), short fifth finger (HP:0009237), and hirsutism (HP:0001007)(Kline et al., 2018). At that time, molecular diagnosis was performed by sequencing the *NIPBL* gene and a potential disease‐causing variant was identified [*NIPBL*:NM_133433.3:c.7553A>G, p.(Asp2518Gly)]. The variant, that induces changes in the surface charge of the protein (Figure 1c), was classified as likely pathogenic (PM2, PP2, and PP3) according to the ACMG/AMP 2015 guidelines (Richards et al., 2015). Segregation studies revealed that the patient's mother (II.4), one aunt (II.2), and one uncle (II.1) carried the variant. The mother (II.4) was clinically evaluated and dysmorphic facial features fully consistent with her daughter's phenotype were observed. Facial photographs of individuals II.2, II.3, and I.2 were available and checked with the Face2Gene application (Latorre‐Pellicer et al., 2020). A medium‐low probability of CdLS was assigned for the aunt (II.2) that was the only one with the c.7553A>G *NIPBL* variant (Figure 1a). Altogether, clinical and molecular findings supported the CdLS diagnosis in the patient.

However, clinical follow‐up of the proband revealed an evolution from CdLS to KBGS features. At the age of six, the patient showed an evident KBGS gestalt with macrodontia (HP:0001572), triangular face (HP:0000325), or bulbous nasal tip (HP:0000414). An additional clinical analysis was carried out with Face2Gene. At 3 years old, KBGS and CdLS were the first and second syndromes suggested, respectively, whereas at 6 years old, CdLS diagnosis did not appear between the top‐5 diagnosis provided by Face2Gene (Figure 1b). A molecular diagnosis reevaluation was performed by using a targeted gene panel including *ANKRD11*, in which a pathogenic nonsense variant was identified [*ANKRD11*:NM_001256183.1:c.2512C>T, p.(Arg838*)] (Figure 1d). This variant was maternally inherited, and neither the aunts (II.2 and II.3) nor the uncle (II.1) had it (Figure 1a). The variant was classified as pathogenic (PVS1, PM2, PP3, and PP5).

Considerable efforts have been made to standardize the interpretation of genetic variants in the laboratory (Latorre‐Pellicer et al., 2020). However, once again, a clear example of the existing limitations is shown here, reinforcing the relevance of the implementation of protocols for periodic reevaluation of phenotypic and genetic information in clinical laboratories. Furthermore, this case highlights the clinical challenges in interpreting multiple pathogenic variants in single patients. An incorrect genetic diagnosis can have severe consequences for prognostic and therapeutic management of the patient, and, as in this case, for the reproductive advice. Moreover, our findings seem to support recent evidences of age‐dependent phenotypic evolution in individuals harboring *ANKRD11* variants (Parenti et al., 2021), and demonstrate the importance of including KBGS in the differential diagnosis of young children with CdLS features. Therefore, it is crucial to include *ANKRD11* in gene panels used for molecular testing of individuals presenting with clinical characteristics of CdLS.

## Data Availability

The data that support the findings of this study are available from the corresponding authors upon reasonable request.
